# A novel nanobody broadly neutralizes SARS‐CoV‐2 via induction of spike trimer dimers conformation

**DOI:** 10.1002/EXP.20230086

**Published:** 2023-12-15

**Authors:** Yang Yang, Junfang Zhang, Shengnan Zhang, Chenhui Zhang, Chenguang Shen, Shuo Song, Yanqun Wang, Yun Peng, Xiaohua Gong, Jun Dai, Chongwei Xie, Tatyana Aleksandrovna Khrustaleva, Vladislav Victorovich Khrustalev, Yongting Huo, Di Lu, Da Yao, Jincun Zhao, Yingxia Liu, Hongzhou Lu

**Affiliations:** ^1^ Shenzhen Key Laboratory of Pathogen and Immunity Shenzhen Clinical Research Center for infectious disease Shenzhen Third People's Hospital Second Hospital Affiliated to Southern University of Science and Technology Shenzhen China; ^2^ Medical Research Center Yuebei People's Hospital, Shantou University Medical College Shaoguan China; ^3^ State Key Laboratory of Respiratory Disease National Clinical Researcher Center for Respiratory Diseases Guangzhou Institute of Respiratory Health The First Affiliated Hospital of Guangzhou Medical University Guangzhou Guangdong China; ^4^ BSL‐3 Laboratory (Guangdong) Guangdong Provincial Key Laboratory of Tropical Disease Research School of Public Health Department of Laboratory Medicine Zhujiang Hospital Southern Medical University Guangzhou China; ^5^ Health and Quarantine Laboratory Guangzhou Customs District Technology Centre Guangzhou Guangdong China; ^6^ Shenzhen Immunity Biotech Co., Ltd. Shenzhen China; ^7^ Multidisciplinary Diagnostic Laboratory Institute of Physiology of the National Academy of Sciences of Belarus Minsk Belarus; ^8^ Guangdong Fapon Biopharma Inc. Shenzhen China; ^9^ Department of Thoracic Surgery The First Affiliated Hospital of Shenzhen University Shenzhen Second People's Hospital Shenzhen Guangdong China

**Keywords:** broad neutralizing, inhalable administration, nanobody, SARS‐CoV‐2, spike trimer dimers

## Abstract

The ongoing mutations of the SARS‐CoV‐2 pose serious challenges to the efficacy of the available antiviral drugs, and new drugs with fantastic efficacy are always deserved investigation. Here, a nanobody called IBT‐CoV144 is reported, which exhibits broad neutralizing activity against SARS‐CoV‐2 by inducing the conformation of spike trimer dimers. IBT‐CoV144 was isolated from an immunized alpaca using the RBD of wild‐type SARS‐CoV‐2, and it showed strong cross‐reactive binding and neutralizing potency against diverse SARS‐CoV‐2 variants, including Omicron subvariants. Moreover, the prophylactically and therapeutically intranasal administration of IBT‐CoV144 confers fantastic protective efficacy against the challenge of Omicron BA.1 variant in BALB/c mice model. The structure analysis of the complex between spike (S) protein, conducted using Cryo‐EM, revealed a special conformation known as the trimer dimers. This conformation is formed by two trimers, with six RBDs in the “up” state and bound by six VHHs. IBT‐CoV144 binds to the lateral region of the RBD on the S protein, facilitating the aggregation of S proteins. This aggregation results in steric hindrance, which disrupts the recognition of the virus by ACE2 on host cells. The discovery of IBT‐CoV144 will provide valuable insights for the development of advanced therapeutics and the design of next‐generation vaccines.

## Graphical abstract

This study describes a novel nanobody that could induce the trimer dimers conformation of the Spike protein to neutralize the SARS‐CoV‐2 infection. This nanobody exhibits a broad neutralizing spectrum against various SARS‐CoV‐2 variants and has the potential to be administered through inhalation.

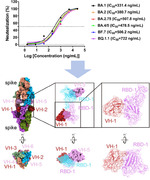

## 1 INTRODUCTION

Although over three years have passed since its emergence, the pandemic of severe acute respiratory syndrome coronavirus 2 (SARS‐CoV‐2) has now been ongoing, causing a devastating burden to global health and economy. Remarkable progress in the vaccines and antiviral drugs against SARS‐CoV‐2 has been achieved,^[^ ^1^ ^]^ while the ongoing mutations of the viruses pose serious challenges to the efficacy of the available antiviral drugs against SARS‐CoV‐2. Therefore, novel drugs with fantastic efficacy are always deserved investigation. Monoclonal antibodies (mAbs) are appealing as potential therapeutics and prophylactics for viral infections owing to their high specificity, strong neutralizing activity and their ability to enhance immune responses.^[^ ^2^ ^]^ Until recently, an impressive number of mAbs targeting the receptor binding domain (RBD) of SARS‐CoV‐2 have been isolated and characterized, which could be further categorized into four classes based on the targeting epitope landscape.^[^ ^2^, ^3^ ^]^ More than 20 SARS‐CoV‐2 mAbs have entered clinical development and several of these mAbs have received emergency use authorization (EUA) from the FDA and other regulatory agencies worldwide,^[^ ^2^ ^]^ while the clinical application might be largely restricted by the cost and time‐consuming processes of production.^[^ ^4^ ^]^ Moreover, recent studies have shown that Omicron sub‐variants could escape most of the existing mAbs,^[^ ^5^ ^]^ indicating the need for the identification of new neutralizing antibodies.

In addition to the conventional mAbs derived from mice and humans, camel‐derived single‐domain antibodies, which contain only two heavy chains, can sever as a significant alternative for therapeutic antibodies.^[^ ^6^ ^]^ Each heavy chain contains only one variable domain of heavy chain of heavy‐chain (VHH) and CH2, CH3 region, with the natural absence of light chains compared to other antibodies. This type of antibody retains similar or even higher specificity and affinity to traditional antibodies while possessing advantages like small size, high stability, easy access to the target organs and bioengineering potential, absence of Fc‐mediated immune activation, and prokaryotic production with the high amount but low cost.^[^ ^6^ ^]^ Therefore, these antibodies are promising options to fight against COVID‐19, demonstrating satisfactory efficacy. Here, we present the characterization and molecular determination of an inhalable nanobody (NAb) called IBT‐CoV144, which exhibits potent neutralizing activity against a wide range of emerging SARS‐CoV‐2 variants.

## 2 RESULTS AND DISCUSSION

### 2.1 Characterization of the nanobody against circulating variants of SARS‐CoV‐2

**FIGURE 1:**
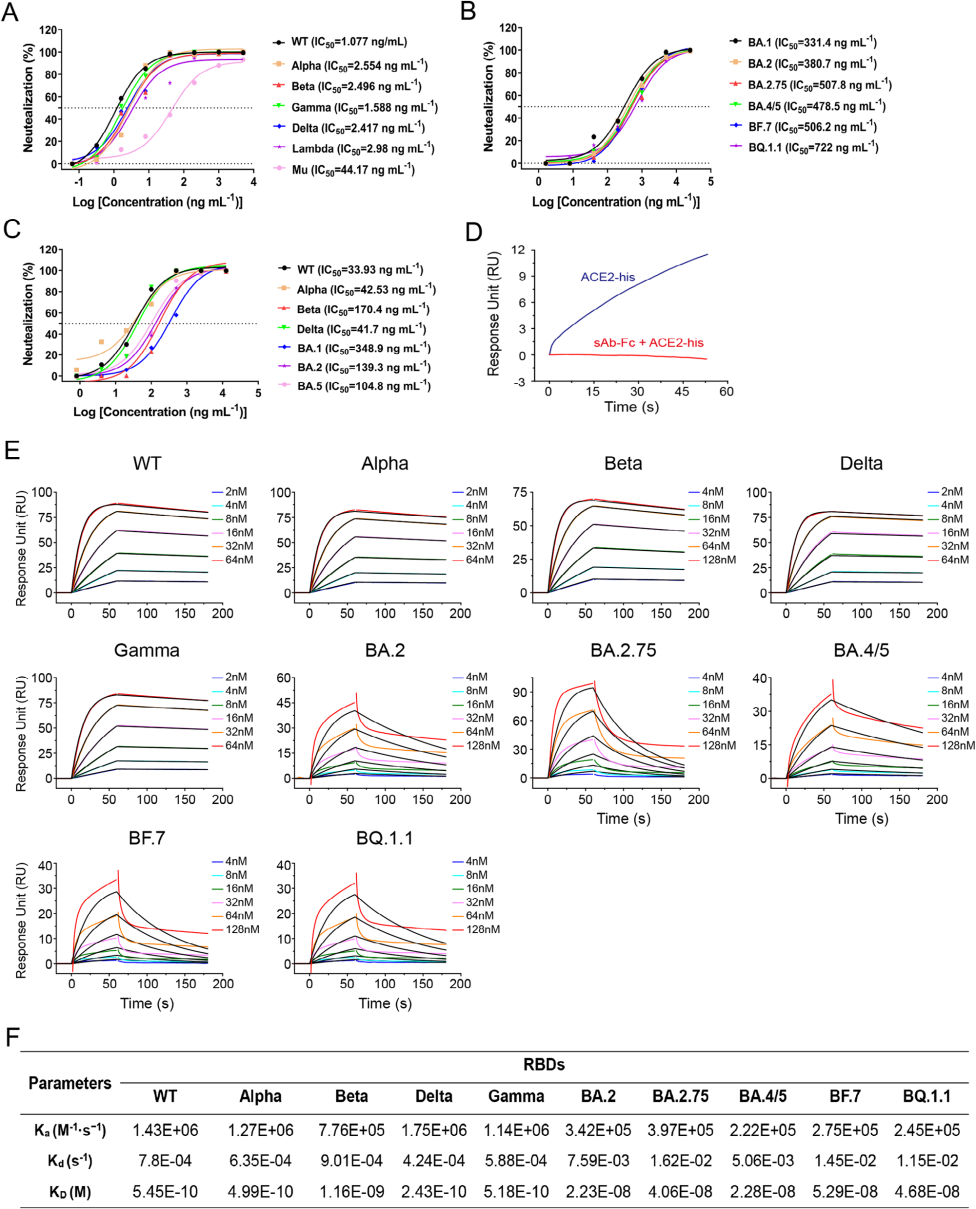
Characterization of binding and neutralizing activities of IBT‐CoV144 against SARS‐COV‐2. Neutralization activity of IBT‐CoV144 against pseudotyped SARS‐CoV‐2 of (A) WT, Alpha, Beta, Gamma Delta, Lambda and Mu virus strains, and (B) Omicron BA.1, BA.2, BA.2.75, BA.4/5, BF.7 and BQ.1.1 virus strains. (C) Identification of neutralizing titer of IBT‐CoV144 using the authentic SARS‐CoV‐2 strains of WT, Alpha, Beta, Delta, BA.1, BA.2 and BA.5. (D) Competition binding to the SARS‐CoV‐2 RBD between IBT‐CoV144 and ACE2 were measured by BLI. The grams show binding patterns after antibody saturation. (E) The binding kinetics between IBT‐CoV144 and RBD proteins from distinct SARS‐CoV‐2 strains were measured by BLI. (F) The *K_a_*, *K_d_* and *K_D_* values of the binding between IBT‐CoV144 and distinct RBD proteins.

A series of NAbs were successfully isolated from the immunized alpacas, and the nanobody named IBT‐CoV144 with excellent binding activity against WT RBD was further analyzed in detail in this study. Firstly, the neutralizing activity of IBT‐CoV144 was analyzed using the pseudotyped SARS‐CoV‐2 viruses (Figure 1A,B). The results showed that this nanobody could efficiently neutralize the variants before Omicron at ultra‐low concentrations varied from 1.077 ng mL^−1^ (WT) to 44.14 ng mL^−1^ (Mu) (Figure 1A), and neutralize the subvariants of Omicron with IC_50_ varied from 331.4 ng mL^−1^ (BA.1) to 722 ng mL^−1^ (BQ.1.1) (Figure 1B). Neutralizing assay using the authentic SARS‐CoV‐2 viruses including WT, Alpha, Beta, Delta, Omicron BA.1, BA.2 and BA.5 variants showed similar results (Figure 1C). Notably, this nanobody could efficiently neutralize the infection of authentic Omicron BA.1, BA.2 and BA.5 variants with IC_50_ of 348.9 ng mL^−1^, 139.3 ng mL^−1^ and 104.8 ng mL^−1^, respectively. The neutralizing ability against Omicron variant was higher than most of the currently available mAbs.^[^ ^5^, ^7^ ^]^ Moreover, we also tested the neutralizing activity of IBT‐CoV144 against seasonal coronaviruses including HCoV‐OC43, HCoV‐NL63 and HCoV‐229E, while no neutralization was found (data not shown). Consistent with the neutralizing assays, this nanobody could bind well with the RBD of WT, Alpha, Beta, Delta and Gamma variants, while showed decreased binding activities to the RBD of BA.2, BA.2.75, BA.4/5, BF.7 and BQ.1.1 variants with K_D_ values varied from 2.23E‐08 to 5.29E‐08 (Figure 1E,F). Then we performed a competition assay using BLI to evaluate whether IBT‐CoV144 could inhibit the binding between RBD and ACE2, and the result showed that this nanobody could compete with ACE2 for binding to the SARS‐CoV‐2 RBD (Figure 1D).

### 2.2 Prophylactic and therapeutic efficacy of IBT‐CoV144 in BALB/c mice against SARS‐CoV‐2

**FIGURE 2:**
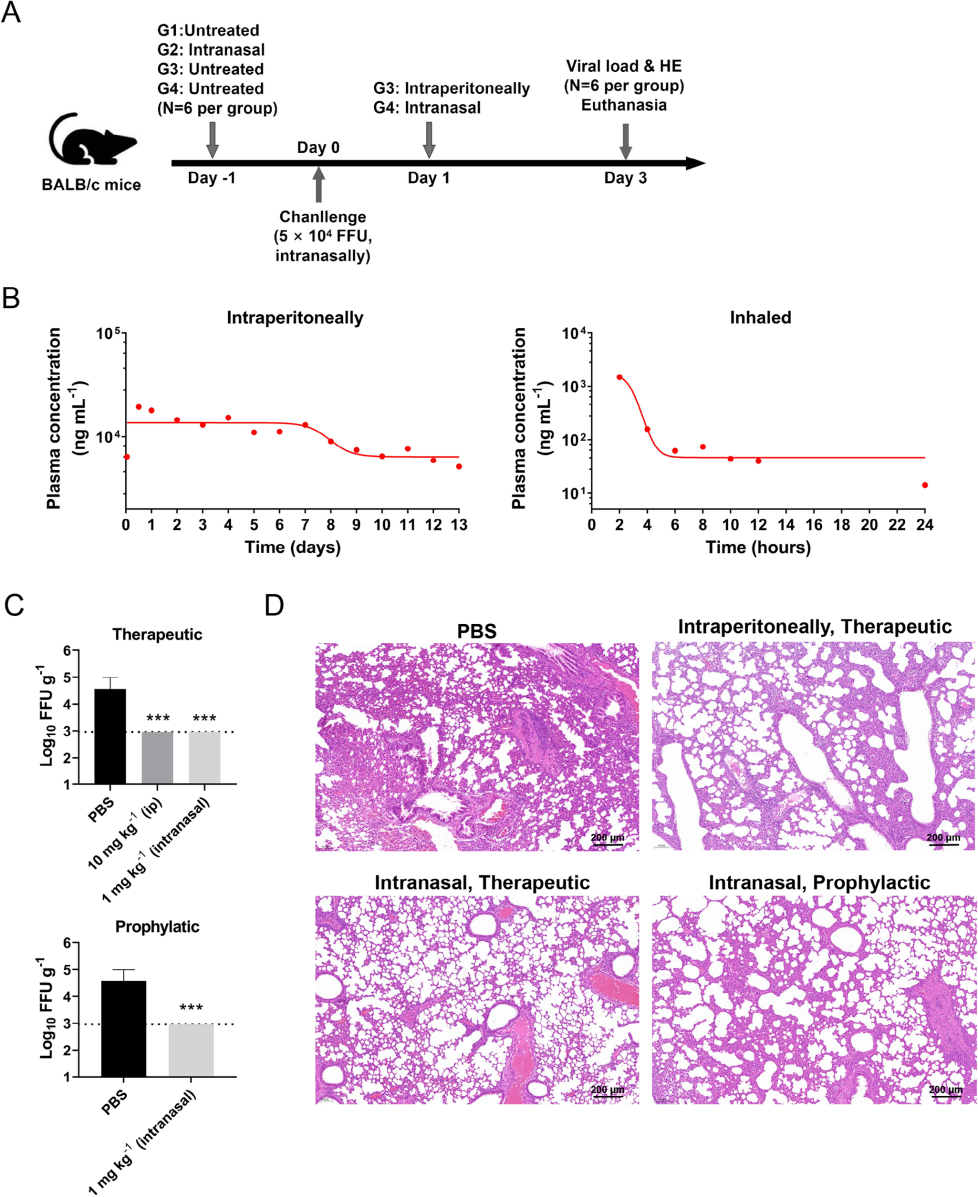
In vivo prophylactic and therapeutic efficacy of IBT‐CoV144 in mice model challenge with SARS‐CoV‐2. (A) Schematic diagram for the evaluation of the antiviral activity of IBT‐CoV144 against the infection of SARS‐CoV‐2 in vivo. (B) The dynamic changes of the concentrations of NAbs in sera samples from treated mice via IP or intranasal. (C) The virus titer in lungs of different groups treated with IBT‐CoV144 (*N* = 6) or PBS (*N* = 6) prophylactically or therapeutically were determined at 2 dpi by the focus forming assay. The NAb treatment group can reduce the viral load in the lung of mice (unpaired *t*‐test, ***p < 0.001). (D) Representative histopathology of the lungs in SARS‐CoV‐2 infected BALB/c mice (2 dpi) treated with PBS or IBT‐CoV144 through different routes of administration.

Firstly, we measured the pharmacokinetics of this NAbs in sera samples from treated mice via intraperitoneal injection (IP) or inhalation. For the IP treatment, the blood concentration of IBT‐CoV144 remained high and stable within four days after injection (d.a.i.), and began to decrease at about 6 d.a.i. Meanwhile, no adverse effects were observed during the 14 days experiment. For the inhalation group, the blood concentration of IBT‐CoV144 decreased dramatically within 6 h after treatment and then remained stable at about 40 ng mL^−1^ within 24 h (Figure 2B). Then the prophylactic and therapeutic efficacy of the nanobody against challenge with Omicron BA.1 were determined using mice model (Figure 2A). To evaluate the therapeutic efficacy of IBT‐CoV144 against the challenge of SARS‐CoV‐2 in vivo, mice were treated with 1 mg kg^−1^ IBT‐CoV144 via inhalation or 10 mg kg^−1^ IBT‐CoV144 via IP at 24 h post challenge. For the prophylactic assay, mice were treated with 1 mg kg^−1^ IBT‐CoV144 via inhalation at 24 h before the virus challenge. The results showed that both prophylactic and therapeutic treatments with this nanobody via IP and inhalation could significantly reduce the viral titers in the lung of the mice when compared to the PBS group (Figure 2C). Moreover, histopathological examination showed bronchopneumonia and interstitial pneumonia in the mice of the PBS group, with a variety of lesions including perivascular to interstitial inflammatory cell infiltrates, necrotic cell debris, and alveolar edema, and the nanobody treatment significantly alleviated these histopathological changes (Figure 2D).

### 2.3 Structural basis for neutralization of IBT‐CoV144

**FIGURE 3:**
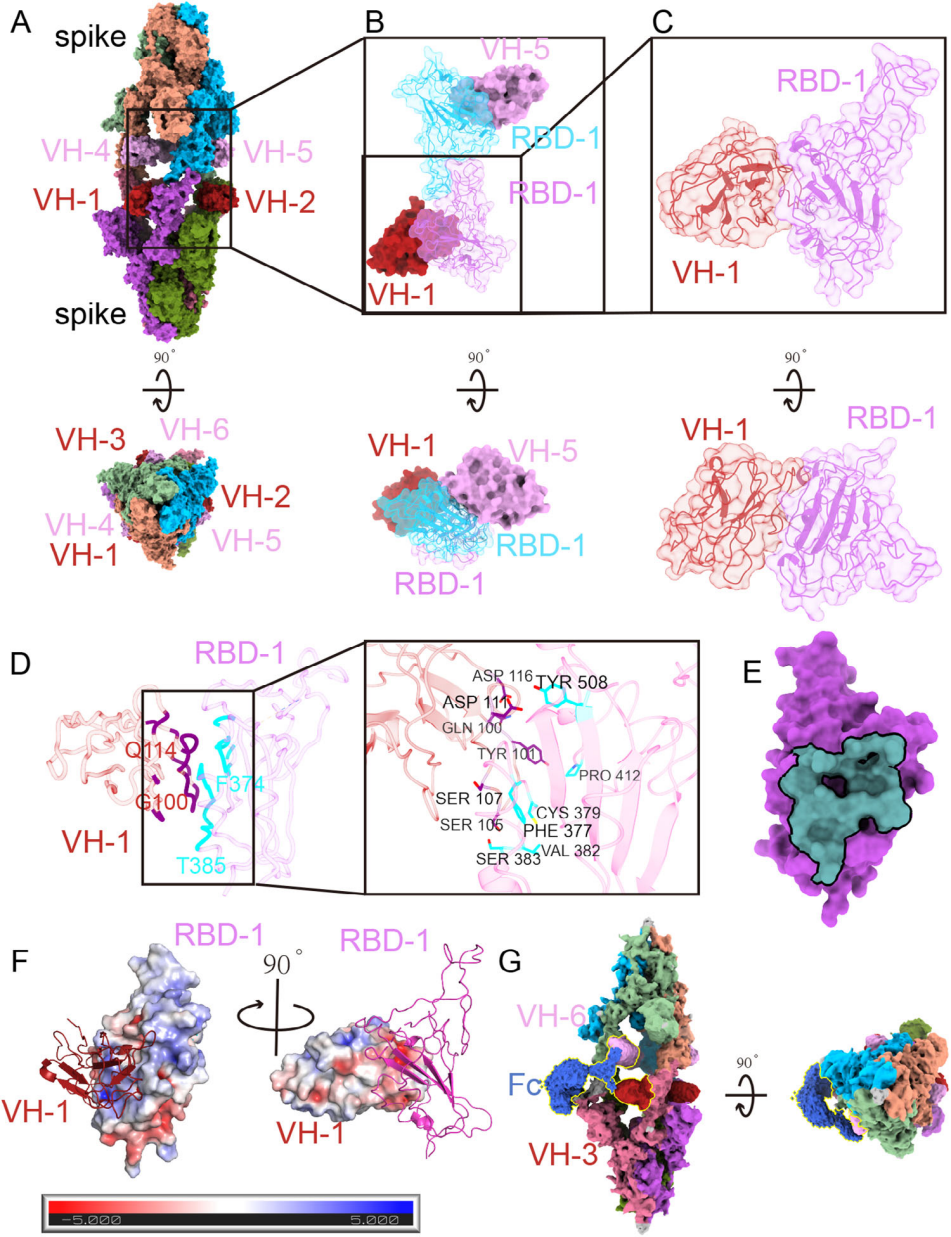
Cryo‐EM structure of Spike‐Nabs complex. (A) Structure of the whole head‐to‐head spike‐Nabs complex. Different spike monomers were colored by olive drab, medium orchid, pale violet red, dark salmon, dark sea green and deep sky blue. The upper VHHs were colored by plum and the nether VHHs were colored by fire brick. The atomic model is shown as the surface and cartoon in ChimeraX. Structure of (B) dimer and (C) monomer of RBD‐VHHs complex. The colors were the same as (A). (D) Interaction amino acids of RBD, colored cyan, and VHH, colored purple. (E) The binding footprint of VHH on RBD. (F) Electrostatics of RBD and VHH were calculated by the APBS electrostatics plugin of Pymol. (G) The C1 symmetry density map. The Fc fragment of the “Y” shaped Nabs is colored blue and the two VHHs were colored plum and fire brick respectively.

The Cryo‐EM structure of the complex between the S protein (BA.2 variant) and IBT‐CoV144 was determined to investigate the neutralization mechanism of this nanobody (Figure 3; Figure S1 and Table S1). The structure was resolved at an overall resolution of 3.96 Å (Figure 3A; Figure S1A). Similar to two previously reported antibodies with comparable neutralizing activity, the Fu2 nanobody and the 6M6 antibody (Figure S5),^[^ ^8^ ^]^ the structure of the spike‐IBT‐CoV144 complex also shows dimerization of the spike trimer. The two trimeric spikes are bound by three full‐length IBT‐CoV144, resulting in a head‐to‐head binding mode. Six RBDs from the two spike trimers are in the “up” state and are bound by six VHHs (Figure 3A). Due to the inherent instability of VHHs and RBDs, a mask was created using the density of the RBD‐VHH dimeric region. Subsequently, local refinement was performed to get a density map with a resolution of 4.05 Å (Figure 3B). The binding of the VHH to the Class 4 epitope was demonstrated, with each VHH binding to a single epitope of the RBD (Figure 3C–E).

The interaction interface between the RBD and VHH was further analyzed (Figure 3D–F). The main amino acids involved in the interaction were found within the F374‐T385 loop of the RBD. Sequence alignment of different variants (WT, Delta, Alpha, Beta, Gamma, BF.7, BQ.1.1, BA.4/5, BA.2, BA.2.75) reveals that 84% (21/25) of the amino acids were conserved (Figure S3). The amino acids F377, C379, V382, S383, P412, and Y508 on the RBD can interact with IBT‐CoV144 by forming hydrogen bonds (Figure 3D; Figure S3). Furthermore, a clear distinction was observed between Gamma and Omicron variants, as the amino acids in the interacting region remained consistent among the WT, Delta, Alpha, Beta and Gamma variants or among Omicron subvariants BF.7, BQ.1.1, BA.4/5, BA.2 and BA.2.75. Among these 25 amino acids in the interacting area, four mutations (S375F, T376A, D405N, and R408S) were analyzed between the non‐Omicron strains and the Omicron strains studied in our research. These specific mutations can potentially alter the surface properties of RBDs, leading to a decrease in neutralizing and binding activities for Omicron subvariants (Figure 1C,F). For instance, the neutral amino acid serine is replaced by phenylalanine, which has an aromatic R group. The polar threonine is substituted with alanine, which has an aliphatic side chain. Additionally, the acidic amino acid aspartic acid is changed to asparagine, which has a neutral R group. Furthermore, the positively charged arginine, with an isoelectric point (PI) of 10.76, mutates to serine, which has a PI of 5.68. However, due to the limitations in the resolution of our map, we are unable to provide further clarity on how exactly these mutations affect the surface properties of RBDs change. To investigate the static potential of RBD and VHH, separate calculations were conducted. The results showed that the VHH at the interface of the interaction carried a negative charge, whereas the RBD had a positive charge. As a result of electrostatic interactions, the two proteins formed a complex (Figure 3F). Additionally, the “Y”‐shaped full‐length antibody can be observed in the C1 symmetry density map, with the Fc fragment positioned along the center of the spike trimer dimers. This observation indicates that VH1 and VH4 are derived from the same nanobody, as are VH2/VH5 and VH3/VH6 (Figure 3G; Figure S4).

Only a few mAbs against the pathogens that induce respiratory tract infection (RTI) have gained the approvals from FDA with the dominant factors of the unsatisfied curative effect and high cost.^[^ ^9^ ^]^ mAbs given through subcutaneous or intravenous administration barely reach the sites of respiratory virus infection due to the air‐blood barrier, which significantly limits therapeutic efficiency.^[^ ^10^ ^]^ Pharmacokinetic studies in primates and neonatal lambs indicated an approximate 500‐ to 2000‐fold lower concentration of mAbs in bronchoalveolar lavage fluid (BALF) than that in plasma.^[^ ^11^ ^]^ Inhalation therapy is a relevant treatment method for pulmonary diseases for their convenience, enhanced bioavailability, rapid onset of action, and reduced systemic exposure.^[^ ^12^ ^]^ Attribute to marked stability and solubility, nanobodies can resist aerosolization for inhalation delivery for the treatment of pulmonary diseases or infections.^[^ ^12^ ^]^ Meanwhile, the small size of nanobodies could facilitate their deposition downward to the middle airways and move to the deep alveolar region by Brownian diffusion.^[^ ^9^ ^]^ These characteristics make nanobodies a promising treatment against SARS‐CoV‐2. At present, several nanobodies against SARS‐CoV‐2 with excellent performance have been reported,^[^ ^13^ ^]^ our nanobody represents a novel one of them with a broad spectrum, novel recognition epitope, and comparable neutralizing activity while the unique mechanism of neutralization.

The Cryo‐EM structure of the spike‐IBT‐CoV144 complex revealed a special conformation of trimer dimers formed by two trimers and three full‐length IBT‐CoV144 NAbs. In this conformation, all six RBDs are in the “up” state and are bound by six VHHs (Figure 3), similar to another NAb named Fu2 (Figure S2).^[^ ^8^ ^]^ Interestingly, the VHH of IBT‐CoV144 bounds to a single epitope of the RBD (Figure 3C–E), while the Fu2 nanobody could recognize two epitopes on the RBD (Figure S2). Regarding the mAb named 6M6, the majority of particles (70%) were trimer consisting of one down‐RBD and two up‐RBDs. Each up RBD was bound to one 6M6 Fab. The remaining particles (30%) exhibited a conformation of trimer dimers.^[^ ^8^ ^]^ In contrast to 6M6, our study revealed that the complex formed by S and IBT‐CoV144 consisted of only one type. Numerous projection images of the complex were collected and no other types of complex configurations were observed during the process of structural analysis. This difference might result from the different sizes, affinity and binding epitopes.^[^ ^14^ ^]^ In addition, the interface area between two RBDs in the RBD‐VHH dimeric portion (456.8 Å^2^) was smaller than that in the RBD‐6M6 dimeric portion (562.7 Å^2^) (Figure 3B; Figure S2), which affected the stability of the RBD‐VHH region. These reasons may contribute to the more flexible state of our complex and also the low resolution of the RBD‐VHH portion in our complex (Figure S1B,C). Furthermore, the structural analysis of the Spike‐CoV‐IBT144 complex provides insights into the neutralizing mechanism. In brief, the IBT‐CoV144s have the ability to bind to the RBD regions on the S protein, thereby stabilizing the RBDs in the spike in the “up” conformation. This prevents the RBDs from attaching to ACE2 receptors on host cells, thus serving a protective function. Additionally, IBT‐CoV144 also facilitates the aggregation of S proteins, leading to steric hindrance that disrupts the virus's recognition ACE2. The combination of these two mechanisms can lead to viral aggregation, obstructing the binding sites of the S protein to ACE2, thereby preventing the initial step of viral invasion into the host.

## 3 CONCLUSION

In summary, we identified and characterized a novel nanobody that could induce the trimer dimers conformation of the Spike protein. This nanobody exhibits a broad neutralizing spectrum against various SARS‐CoV‐2 variants and has the potential to be administered through inhalation. These findings suggest that it could be a promising candidate for a therapeutic drug against the Omicron variant.

## AUTHOR CONTRIBUTIONS

Hongzhou Lu, Jincun Zhao and Yingxia Liu conceived and supervised the study. Jincun Zhao, Chongwei Xie, Yongting Huo, Di Lu, and Da Yao contribute to the isolation of the nanobody. Yang Yang, Shengnan Zhang, Yun Peng, Xiaohua Gong, Yanqun Wang, and Jun Dai contribute to the evaluation of the neutralizing potency using authentic virus in vitro and in vivo. Shuo Song performed the affinity experiments. Tatyana Aleksandrovna Khrustaleva and Vladislav Victorovich Khrustalev helped to polish the language and interpretation of the data. Chenhui Zhang performed cryo‐EM studies. Yang Yang and Chenguang Shen designed the experiments and wrote the manuscript.

## CONFLICT OF INTEREST STATEMENT

The authors declare no conflicts of interest.

## Supporting information

Supporting Information

## Data Availability

All data related to this work are present in the article and in the Supporting Information.
