# Supplementary material for: A novel nanobody broadly neutralizes SARS‐CoV‐2 via induction of spike trimer dimers conformation
Source: Exploration (Beijing). 2023 Dec 15;4(3):20230086. doi: 10.1002/EXP.20230086 (PMC11189563; doi:10.1002/EXP.20230086)
Supplement: Supplementary file 1 — Supporting Information [file EXP2-4-20230086-s001.pdf]

Supporting Information

1. Experimental section

1.1 Cell lines and viruses

Vero cells (ATCC, CCL-81) were obtained from ATCC, and 293 cells stably expressing ACE2 (293-ACE2) were obtained from Vazyme. Clinical isolates of wild-type (WT) SARS-CoV-2 (EPI_ISL_406594), Alpha (EPI_ISL_11799982), Delta (EPI_ISL_11799983), Omicron BA.1 (EPI_ISL_11799984), BA.2 (EPI_ISL_17509571) and BA.5 (EPI_ISL_16968305) were isolated from the respiratory samples of the COVID-19 patients using Vero cell in biosafety level 3 (BSL-3) laboratory. The Beta variant (GDPCC-nCoV84) was kindly provided by the Guangdong Provincial Center for Disease Control and Prevention (CDC), Guangdong Center for Human Pathogen Culture Collection (GDPCC). HCoV-OC43 (ATCC, VR-1558) and HCoV-229E (ATCC, VR-740) were obtained from ATCC, and HCoV-NL63 (Genebank No. JX104161) was a gift from the National Institute for Viral Disease Control and Prevention, China CDC. The 50% tissue culture infectious dose ($TCID_{50}$) assay was done to measure the titers of the viral stocks as previously described[1].

1.2 Generation of RBD proteins of WT SARS-CoV-2 and SARS-CoV-2 variants.

The coding sequences of SARS-CoV-2 spike RBD proteins were achieved from the UniProt website (https://www.uniprot.org/). The RBD proteins of the WT and other variants of SARS-CoV-2 with 10 × his tag at the N terminal were expressed in HEK 293T cell and purified with Ni-NTA affinity columns (Qiagen, Hilden, Germany).

1.3 Alpaca immunization and antibody selection by phage display

Four alpacas were injected with WT SARS-CoV-2 RBD every 7 days for a total of six times, then the peripheral blood samples from these immunized alpacas were collected for the extraction of lymphocyte cells and construction of phage libraries. All procedures were performed according to the Health Guide for the Care and Use of Laboratory Animals.

Biopanning of NAbs against WT RBD was performed, and the positive clones from the libraries were picked and expressed in microplates. The individual colonies were inoculated in 2 mL of LB medium with 100 μg mL^{-1} ampicillin in 24-well deep well plates and cultured for 5 hours at 37°C. Then, isopropyl b-D-1-thiogalactopyranoside (IPTG) (0.2 mM) was added to induce the expression of VHH at 25°C overnight, and the bacterial cells were pelleted and resuspended in 300 μL TES buffer (0.2 M Tris-HCl pH 8, 0.5 mM EDTA, 0.5 M sucrose) and incubated at 4°C for 30 min. Subsequently, 350 μL water was added to induce an osmotic shock. After 1 hour incubation at 4°C, the periplasmic extract was centrifuged and collected. The VHH-containing periplasmic extracts were then tested for binding to SARS-CoV-2 RBD protein by ELISA, and positive clones were subjected the further analysis. After screening, the RBD-specific clones were sequenced and amplified. Then the plasmids of these candidates were transformed into Escherichia coli strain BL21 for expression, and Ni-NTA affinity columns (Qiagen, Hilden, Germany) were used for NAbs purification, and the purified NAbs were confirmed through SDS-PAGE.

1.4 Mammalian protein expression and purification

Mammalian expression plasmids pcDNA3.1-IBT-CoV144 containing 2 VHHs fused with Fc were transfected into FreeStyle293 cells using polyethylenimine (PEI) as previously reported[2]. All of these expression plasmids contained N-terminal signal sequences to ensure the secretion of the VHHS into the cell supernatant. Briefly, suspension-adapted and serum-free FreeStyle293 cells were seeded at 3×10^6 cells mL^{-1} in Freestyle-293 medium (Thermo Fisher Scientific), and 100 mL culture grown at 37C and 8% CO_2 was transfected with 50 μg of pcDNA3.1-VHH-Fc plasmid DNA using PEI. One day after transfection, 3.5 mL of FreeStyle feed was added to the cells, and cultures were further incubated at 32°C with 5% CO_2. Cells were fed a second time at 3 days post-transfection. Then the cultures were harvested as soon as the cell viability dropped below 75%. For purification of the VHH-Fc,

supernatants were loaded on a 5 mL Protein A resin (GE Healthcare, USA). The bound antibodies were eluted using 0.1M glycine buffer (pH 3.0), and then the antibody-containing fractions were neutralized with Tris-Hcl (pH 8.0) immediately after the elution. These neutralized fractions were then pooled, and loaded onto a HiPrep Desalting column for buffer exchange into PBS buffer.

1.5 Affinity determination

The binding kinetics between IBT-CoV144 and SARS-CoV-2 RBD from different variants were assessed using biofilm interferometry (BLI) with a Fortebio's Octet RED96 instrument (ForteBio, Menlo Park, USA). Briefly, the diluted NAbs (10 μg mL^{-1}) were coupled to protein A biosensors and then incubated with serially diluted RBD proteins, followed by dissociation in PBST. The binding curves were fit in a 1:1 binding model by Octet Data Analysis software 9.0. The association and dissociation rates were monitored and the equilibrium dissociation constant (K_D) was determined.

1.6 Cryo-EM sample preparation and data collection

The spike protein from the BA.2 variant (about 1.5 mg mL^{-1}) was incubated with IBT-CoV144 at a mole ratio of 1:3 for 30 minutes on the ice. Subsequently, a 3 μL drop of the complex was applied to the glow-discharged Quantifoil grids (Cu R1.2/1.3, 300 mesh) in the FEI Vitrobot Mark IV. After a 4-second blotting step, the grid was rapidly plunged into liquid ethane and then transferred to liquid nitrogen. The cryo-grids were then transferred to an FEI Titan Krios G3i microscope with the Gatan BioQuantum energy filter connected to Gatan K2 Summit direct electron detector. Cryo-EM data were collected using serialEM software. Each exposure had a total exposure time of 4.43 seconds, with approximately 138 ms per frame, resulting in a 32-frame movie per exposure and an accumulated dose of 50 $e^-/Å^2$. The raw data were saved at the pixel size of 0.421 Å. The defocus range during data collection was set in the range of -1.5 to -2.5 μm. A total of 8,546 movies were collected.

1.7 Cryo-EM data processing and reconstruction

The movies were gain-normalized, binned by 2 with Fourier cropping, and corrected for beam-induced motion using MotionCor2[3]. The CTF parameters were determined by GCTF[4]. After that, 7496 good micrographs were selected based on the estimated CtfMaxResolution value to pick particles with cryoSPARC3.3.2[5] Blob picker. A total of 2,112,915 particles were picked out and extracted as 4× binned particle images (3.368 Å/pixel) using cryoSPARC. After several rounds of 2D classification, the result was used for the template picking using cryoSPARC. A total of 742,674 particles were picked out and extracted as 4 × binned particle images. After another several rounds of 2D classification and removal of duplicates with a minimum separation distance of 60 Å, 192,476 particles were settled. Then, Ab-Initio Reconstruction and Heterogeneous Refinement were performed successively. 166,906 particles were selected to extract 2 × binned particles (1.684 Å/pixel). These particles were imported into Relion3.1.2[6] for Class3D with C1 symmetry, k=6, resulting in a 6.33 Å 90,786 good particles for subsequent processing. Unbinned particles were extracted and Refine3D with C1 or C3 symmetry was performed, resulting in a 4.98 Å density map (C3). After a post-process job with a mask created through the refined map by Relion, a map with a resolution of 3.96 Å was acquired. However, due to the flexibility of the RBD and NAb interacting zone, the resolution for this part is lower than the rest part of the complex. To improve the resolution for this region, the particle subtraction job was performed with a mask of the dimer of the RBD-VHH zone, resulting in 272,358 particles. These particles were imported into cryoSPARC for the 3D Classification (BETA) job with 6 classes, using PCA initialization mode. A total of 203,158 particles were selected for local refinement, resulting in a 4.05 Å density map. The local resolution map was calculated by CRYOSPARC. Detailed elaboration for this data processing workflow was shown in Figure S1.

1.8 Model building and refinement

The SARS-CoV-2 BA.2 spike protein (PDB:7XIW, chain D) was used as the initial model to build an atomic model for the spike protein. The initial model for the VHHs was acquired by predicting methods using the SWISS-MODEL (https://swissmodel.expasy.org/). Chain D of 7XIW was fitted into the dimer of the spike-NAbs complex using UCSF Chimera[7]. After that, the atomic model was manually adjusted in COOT[8] and further refined in Phenix using real-space refinement[9]. Repeat the manual adjustment and real space refinement for several rounds, and finally, the coordinates were evaluated by MolProbity[10]. The model refinement statistics are summarized in Supplementary Table S1. The structure figures were prepared using PyMol (http://www.pymol.org) or ChimeraX[10].

1.9 Viral entry inhibition assay with pseudotyped SARS-CoV-2 viruses

SARS-CoV-2 pseudotyped viruses neutralization assays were conducted to test the neutralizing activities of these nanobodies against WT, Alpha, Beta, Gamma, Delta, Lambda, Mu and Omicron variants (Vazyme) as previously reported with little modification [1] [11]. In brief, IBT-CoV144 was serially diluted in 5-folds with DMEM supplemented with 10% FBS in flat-bottom 96-well plates (Thermo Fisher) in a total volume of 50 μL. Then SARS-CoV-2 pseudotyped particles with a concentration of 200 $TCID_{50}$ in an equal volume were added to each well and incubated at 37°C for 1h. After incubation, 4×10^5 293T-ACE2 cells in 100 μL complete media were added per well and incubated for another 48h at 37°C and 5% CO_2. Then, the firefly luciferase activity (luminescence) was measured, and the neutralizing titers were calculated as a 50% inhibitory concentration (IC_{50}), which expressed as the concentrations of nanobodies that resulted in a 50% reduction of luciferase luminescence compared with virus control in single-round-pseudovirus infection assay.

1.10 Authentic SARS-CoV-2 neutralizing assays

Briefly, IBT-CoV144 was serially diluted in 5-folds with DMEM supplemented with 2% FBS in a total volume of 100 μL, then live SARS-CoV-2 was added and incubated at 37°C for 1h

with a concentration of 400 $TCID_{50}$ in 100 μL. After incubation, the mixture was added to the cell in 24-well plates (Thermo Fisher) and incubated for another 1h at 37°C. Then, the virus-drug mixture was removed and cells were further cultured with fresh DMEM with 2% FBS and the indicated concentrations of nanobodies. The cell supernatant was collected and viral RNAs were extracted using the QIAamp RNA Viral Kit (Qiagen, Heiden, Germany) at 48 hours post-infection (h.p.i), and quantitative reverse transcription polymerase chain reaction (qRT-PCR) was performed using a commercial kit (Mabsky Bio-tech Co., Ltd.) to evaluate the neutralization efficiency. All the experiments involving infectious SARS-CoV-2 were handled in BSL-3 facilities at the Shenzhen Third People's Hospital.

1.11 Animal experiments

Twenty-four BALB/c mice (6-8 weeks old) were divided into four groups of six mice each. For the prophylactic and therapeutic groups, mice were treated with IBT-CoV144 intraperitoneally at 10 mg kg^{-1} or through inhalation at 1 mg kg^{-1} 24h before or after the challenge with 5×10^4 FFU SARS-CoV-2 (Omicron BA.1). Control mice were injected with an equal volume of PBS in the same manner as previously reported[12]. To investigate the protective efficacy of IBT-CoV144, all the mice were euthanized at 2 dpi and lungs were harvested for viral titer by using the focus forming assay (FFA), and lung tissues were collected and stained for histopathological analysis, as previously reported[13]. All work with SARS-CoV-2 was conducted in the BSL-3 Laboratories of Guangzhou Customs District Technology Center. The protocol was approved by the Laboratory Animal Ethics Committee of Affiliated First Hospital of Guangzhou Medical University (approval number: 20230671).

4.12 Statistical Analysis

The Mann-Whitney U test was used to determine the differences between groups using GraphPad Prism. P values between 0.01-0.05, 0.001-0.01 and <0.001 were considered statistically significant (*), very significant (**) and extremely significant (***), respectively.

2. Supplementary figures

Figure S1: cryo-EM data processing. (A): Workflow for spike-VH complex dataset processing. (B): FSC curve and (C): local resolution estimation for RBD-VH local refinement.

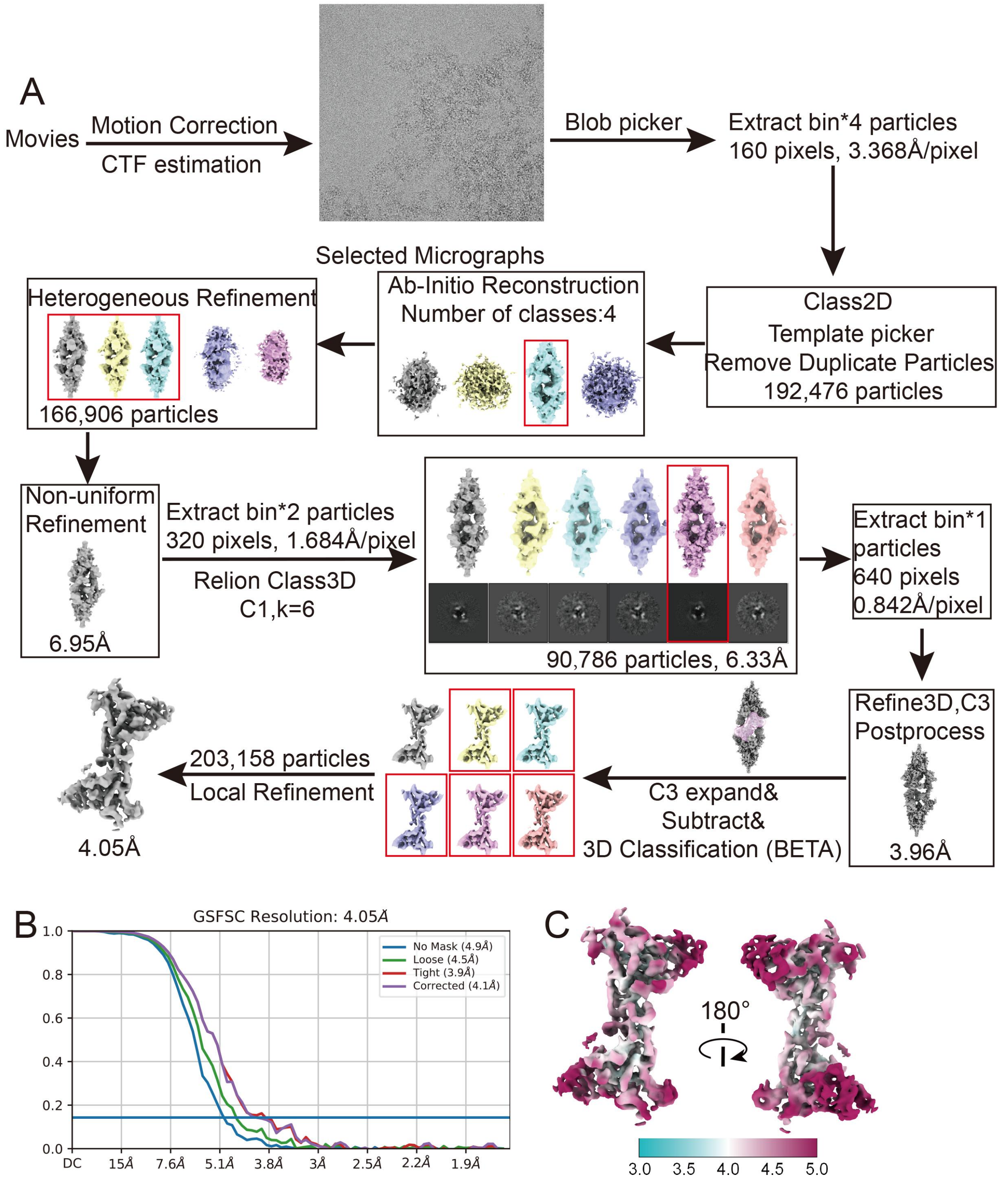

Figure S2: Comparison of the spike-Abs complex map among IBT-CoV144, Fu2 and 6M6.

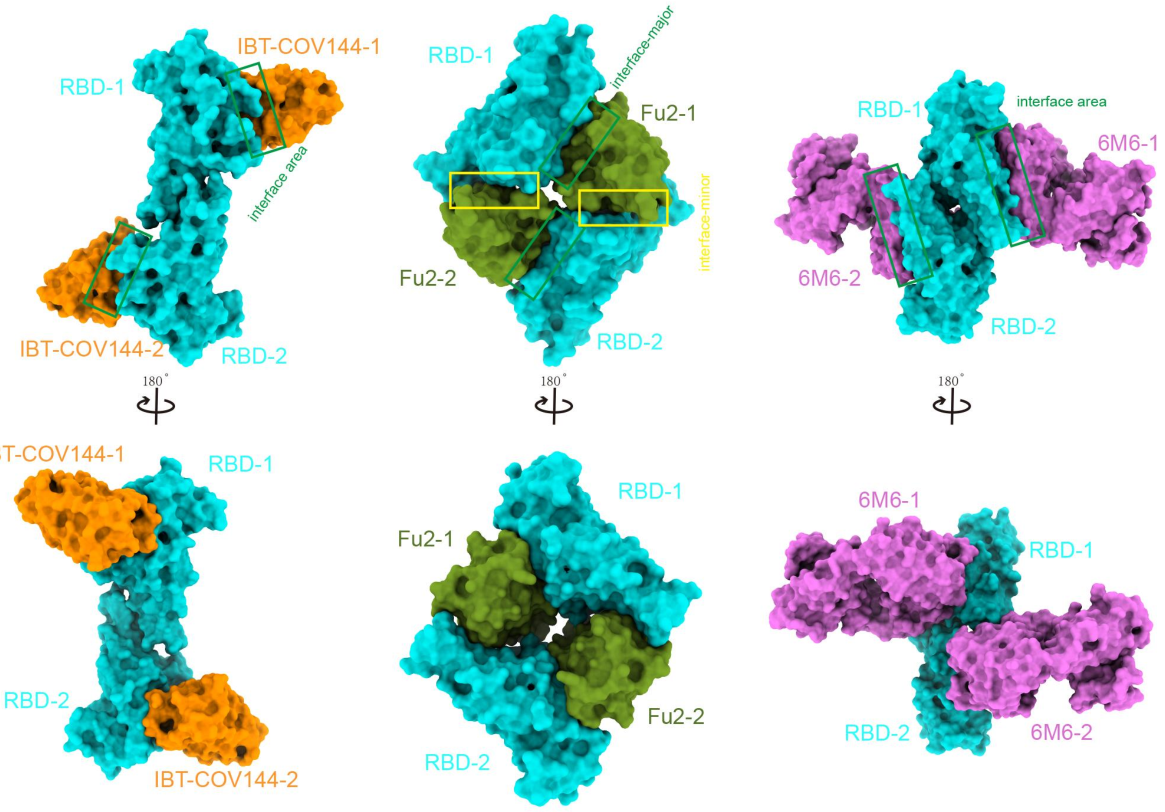

Figure S3: Sequence alignment of different virus strains. The parts that are framed in the green box stand for the amino acids in the interaction area. Black lines between "Gamma" and "BF.7" divide the virus strains into two main types, Omicron subvariants and non-Omicron subvariants. Residues, which may form hydrogen bonds or salt bridges with IBT-CoV144 are highlighted by asterisk

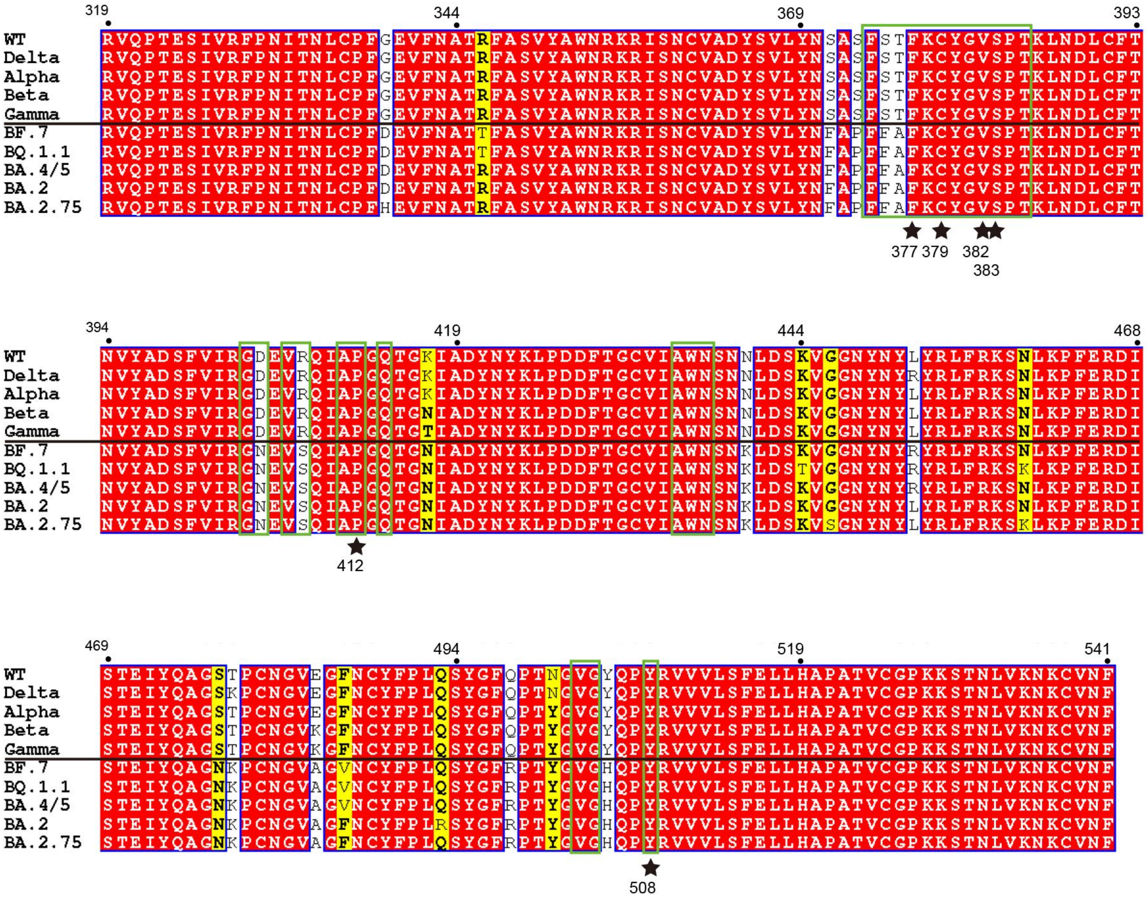

Figure S4: Standard planes of the spike-NAbs complex map. This map was refined with C1 symmetry. There are two "Y" shaped NAbs that can be observed and they are framed out by yellow and red boxes on planes 298 and 313, respectively in Chimera. Plane 270 and 341 shows C3 symmetry since these two planes are parts of the spike trimers.

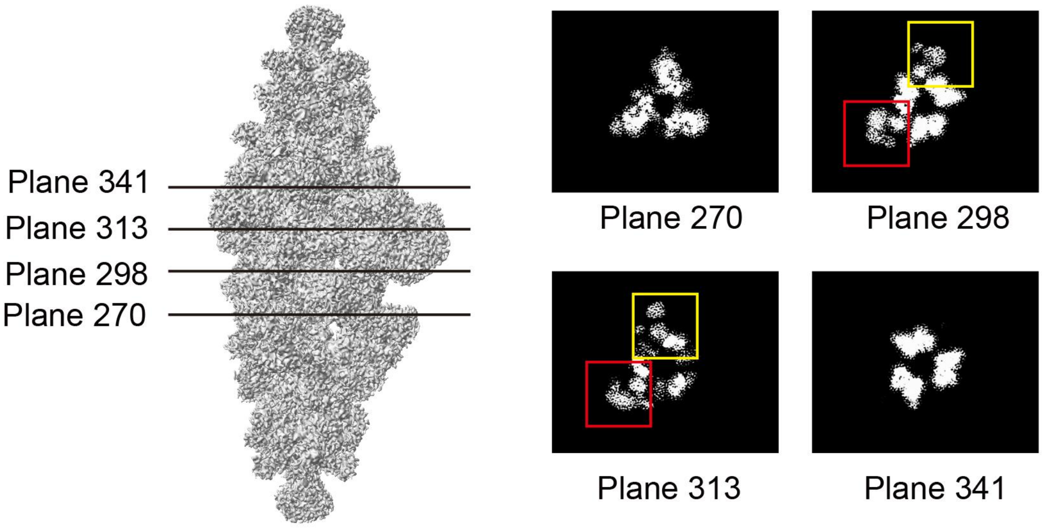

Figure S5: Interaction area between the two RBDs of the dimer of (A) RBDs-VHHs and **(B)** RBDs-6M6 complex. Red or green balls stand for the amino acids in the upper or nether RBD, and blue or cyan balls stand for the surface amino acids in the upper or nether RBD.

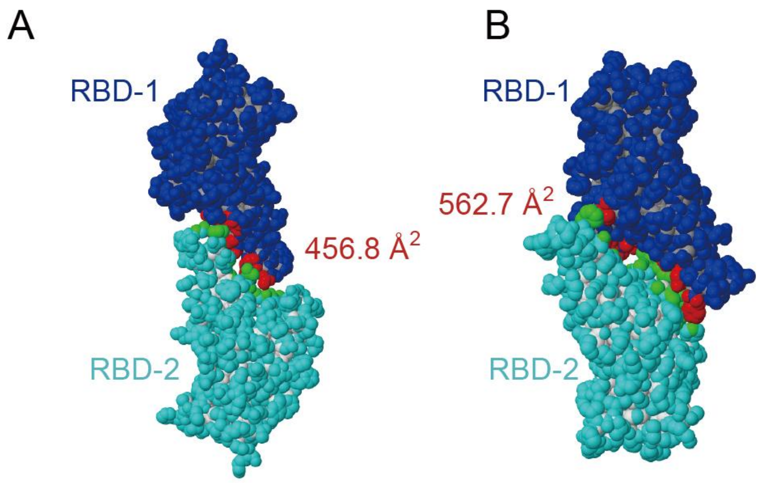

3. Supplementary tables

Table S1. Cyro-EM data collection and model refinement statistics.

**Data collection: Complex of SARS-CoV-2 BA.2 spike and IBT-CoV144**			
Electron microscope	Titan Krios G3		
Camera	K2		
Voltage (kV)	300		
Collection mode	Counting and Super-resolution		
Pixel size(Å/pixel)	0.842		
Total exposure(e-/ Å2)	50		
Number of frames	32		
Defocus range(μm)	-1.5~-2.5		
**Image processing&3D Refinement**			
Motion correction software	MontionCor2		
CTF estimation software	CTFFIND4		
Particle selection software	CryoSPARC 3.3.1		
Micrographs used	8546		
Density map	Dimer of RBD and VHH complex	Dimer of Spike and IBT-CoV144(C3)	Dimer of Spike and IBT-CoV144(C1)
Particles number	203,158	90786	90786
Applied symmetry	C1	C3	C1
Global resolution (FSC=0.143)	4.1	3.96	4.4
EMDB	EMD-36730	EMD-36735	EMD-36740
**Model building and refinement**			
Modeling software	COOT, Chimera	--	--
Protein residue number	441	--	--
MolProbity score	2.65	--	--
Clash score	20.35	--	--
Ramachandaran plot (%)		--	--
Outliers	1.18	--	--
Allowed	12.71	--	--
Favored	86.12	--	--
PDB	8JYS		

Reference

[1] Y. Yang, M. Yang, Y. Peng, Y. Liang, J. Wei, L. Xing, L. Guo, X. Li, J. Li, J. Wang, M. Li, Z. Xu, M. Zhang, F. Wang, Y. Shi, J. Yuan, Y. Liu, *Nature Microbiology* **2022**, 7, 423.

[2] N. S. Laursen, R. H. E. Friesen, X. Zhu, M. Jongeneelen, S. Blokland, J. Vermond, A. van Eijgen, C. Tang, H. van Diepen, G. Obmolova, M. van der Neut Kolfschoten, D. Zuijdgeest, R. Straetemans, R. M. B. Hoffman, T. Nieusma, J. Pallesen, H. L. Turner, S. M. Bernard, A. B. Ward, J. Luo, L. L. M. Poon, A. P. Tretiakova, J. M. Wilson, M. P. Limberis, R. Vogels, B. Brandenburg, J. A. Kolkman, I. A. Wilson, *Science (New York, N.Y.)* **2018**, 362, 598.

[3] S. Q. Zheng, E. Palovcak, J.-P. Armache, K. A. Verba, Y. Cheng, D. A. Agard, *Nature Methods* **2017**, 14, 331.

[4] K. Zhang, *Journal of Structural Biology* **2016**, 193, 1.

[5] A. Punjani, J. L. Rubinstein, D. J. Fleet, M. A. Brubaker, *Nature Methods* **2017**, 14, 290.

[6] J. Zivanov, T. Nakane, B. r. O. Forsberg, D. Kimanius, W. J. H. Hagen, E. Lindahl, S. H. W. Scheres, *eLife* **2018**, 7, e42166.

[7] E. F. Pettersen, T. D. Goddard, C. C. Huang, G. S. Couch, D. M. Greenblatt, E. C. Meng, T. E. Ferrin, *Journal of Computational Chemistry* **2004**, 25, 1605.

[8] P. Emsley, B. Lohkamp, W. G. Scott, K. Cowtan, *Acta Crystallographica Section D* **2010**, 66, 486.

[9] P. D. Adams, P. V. Afonine, G. Bunkoczi, V. B. Chen, I. W. Davis, N. Echols, J. J. Headd, L.-W. Hung, G. J. Kapral, R. W. Grosse-Kunstleve, A. J. McCoy, N. W. Moriarty, R. Oeffner, R. J. Read, D. C. Richardson, J. S. Richardson, T. C. Terwilliger, P. H. Zwart, *Acta Crystallographica Section D* **2010**, 66, 213.

[10] V. B. Chen, W. B. Arendall, III, J. J. Headd, D. A. Keedy, R. M. Immormino, G. J. Kapral, L. W. Murray, J. S. Richardson, D. C. Richardson, *Acta Crystallographica Section D* **2010**, 66, 12.

[11] Y. Yang, X. Gong, J. Wang, S. Fang, J. Zhang, X. Liao, Y. Guan, W. Wu, Y. Liu, H. Lu, *Signal transduction and targeted therapy* **2022**, 7, 316.

[12] a)Y. Wu, F. Wang, C. Shen, W. Peng, D. Li, C. Zhao, Z. Li, S. Li, Y. Bi, Y. Yang, Y. Gong, H. Xiao, Z. Fan, S. Tan, G. Wu, W. Tan, X. Lu, C. Fan, Q. Wang, Y. Liu, C. Zhang, J. Qi, G. F. Gao, F. Gao, L. Liu, *Science (New York, N.Y.)* **2020**, 368, 1274; b)C. Li, W. Zhan, Z. Yang, C. Tu, G. Hu, X. Zhang, W. Song, S. Du, Y. Zhu, K. Huang, Y. Kong, M. Zhang, Q. Mao, X. Gu, Y. Zhang, Y. Xie, Q. Deng, Y. Song, Z. Chen, L. Lu, S. Jiang, Y. Wu, L. Sun, T. Ying, *Cell* **2022**, 185, 1389; c)Y. Wang, M. Liu, Y. Shen, Y. Ma, X. Li, Y. Zhang, M. Liu, X. L. Yang, J. Chen, R. Yan, D. Luan, Y. Wang, Y. Chen, Q. Wang, H. Lin, Y. Li, K. Wu, T. Zhu, J. Zhao, H. Lu, Y. Wen, S. Jiang, F. Wu, Q. Zhou, Z. L. Shi, J. Huang, *Cell discovery* **2022**, 8, 36.

[13] J. Sun, Z. Zhuang, J. Zheng, K. Li, R. L.-Y. Wong, D. Liu, J. Huang, J. He, A. Zhu, J. Zhao, X. Li, Y. Xi, R. Chen, A. N. Alshukairi, Z. Chen, Z. Zhang, C. Chen, X. Huang, F. Li, X. Lai, D. Chen, L. Wen, J. Zhuo, Y. Zhang, Y. Wang, S. Huang, J. Dai, Y. Shi, K. Zheng, M. R. Leidinger, J. Chen, Y. Li, N. Zhong, D. K. Meyerholz, P. B. McCray, Jr., S. Perlman, J. Zhao, *Cell* **2020**, 182, 734.
